# 3D octopus kinematics of complex postures: Translation to long, thin, soft devices and their potential for clinical use

**DOI:** 10.1371/journal.pone.0303608

**Published:** 2024-05-29

**Authors:** Garrett Weidig, Brittany Bush, Fermin Jimenez, Galit Pelled, Tamara Reid Bush

**Affiliations:** 1 Mechanical Engineering Department, Michigan State University, East Lansing, MI, United States of America; 2 Department of Radiology, Michigan State University, East Lansing, MI, United States of America; University of California Irvine, UNITED STATES

## Abstract

**Intro/Background:**

Octopuses are capable of complex arm movements. Unfortunately, experimental barriers and lack of a robust analysis method made it difficult to quantify the three-dimensional (3D) kinematics of soft, flexible bodies, such as the octopus arm. This information is not only crucial for understanding the posture of the animal’s arm but also for the development of similarly designed soft, flexible devices.

**Obj/Goal:**

The primary goal of this work was to create a method to comprehensively quantify complex, 3D postures of octopus (*Octopus Bimaculoides*) arms in a manner that is conducive and translatable to octopus arm-inspired devices for health monitoring and rehabilitation.

**Methods:**

In this study, 3D underwater motion capture was used to collect kinematic data on both live octopuses and disembodied arms that still had neural activity. A new method was developed to define how arm curvature and regional segments were oriented relative to each other in 3D. This included identification of the bend within a segment along with the computation of the relative orientation between segments, thus permitting the complete quantification of complex arm motions.

**Results:**

By comparing vector-based and radius of curvature-based approaches to magnitude of curvature, it was clear that the vector-based approach was less dependent on the length of a segment and that its reported ranges of motion were translatable for outcome measures associated with clinical use. The new approach for the relative orientation of each segment of the octopus arm resulted in the capability of describing the octopus arm in many unique postures, such as straight, simple bending, and complex bending as it utilized the three rotational angles.

**Outcome/Impact:**

This method and its application to octopus arms will yield new information that can be used to better communicate and track not only octopus arm movements but in the development of complex, segmented, soft-bodied devices that can be used in health monitoring and rehabilitation.

## Introduction

The octopus has a unique anatomical structure that results in arms with nearly infinite degrees of freedom [1]. Octopuses, among other anatomically similar structures such as elephant trunks and human tongues, are muscular hydrostats, meaning that their head and arms are comprised largely of nerve, muscle, and connective tissue [2, 3]. There are three primary muscle groups surrounding the axial nerve cord in each octopus arm: they are oriented longitudinally, transversely, and obliquely (connects longitudinal and transverse muscles) [4]. The pairing of the flexible nerve cord and intertwined muscle groups allow for the ability to create a ‘joint’ anywhere along the length of the arm and, thus, are capable of unique kinematics. These movements include bending (curvature induced along the longitudinal axis) in multiple directions and twisting (rotation about the longitudinal axis). Both of which will be the focus of this study as they are directly translatable to soft, wearable devices [1, 2].

Tracking octopus arm movements in experimental settings varied in complexity depending on the objective of the research [1–10]. Some studies interested in the mechanical abilities and range of motion of the octopus observed their behavior and reported how frequently bend and twist motions occurred for certain arm segments (proximal, middle, and distal) [4, 6]. Since data were collected observationally, they only required 2D videotaping of the animals. Other researchers, who aimed to quantify specific movements, relied on more complex approaches to movement tracking [1, 7, 10, 11]. One study investigated arm posture during searching activities but constrained movement to a 2D plane [10]. Although researchers still relied on 2D video cameras, machine learning algorithms were used to track certain points on the arm and those data were used to report the amount of curvature along the arm [10]. Researchers who aimed to collect and analyze 3D data did so by extrapolating 3D data from two synced, standard video cameras [1, 7, 11]. They either used manual or machine learning algorithms to track points on the arm in both camera views. By combining the views, 3D octopus arm reconstructions were created and kinematic analyses like bend and twist calculations were conducted [1, 7, 10, 11]. The different approaches for tracking and discussing arm posture had variable utilities. However, studies that utilized 3D data were more conducive to quantitative analyses, as opposed to observations.

Two approaches were used in literature to report the bending of octopus arms. The first approach used two vectors to define an angle of curvature from three consecutive points [1], [10]. The second approach used the radius of curvature, which was defined using the radius of a circle fit to three consecutive points [7]. The approach used in literature depended on the research objectives. In searching movements, researchers used the vector approach to report 2D bend angles along the arm [10]. A similar approach was used to analyze fetching motions. Researchers found that octopuses primarily used a single bend in their arm to fetch prey after grasp; so instead of tracking the whole arm, they focused on the curvature angle of that primary bend [1]. Researchers often used the radius of curvature approach during reaching motions [7], [11]. Stereotypical reach and grasp movements required the octopus to align their arm with the prey, propagate a wave along their arm, and make fine motor adjustments for grasp [7, 11]. Researchers used a radius of curvature to track the progression of the wave throughout the reaching motion [7, 11]. However, there is currently no standard as to which approach should be used in quantifying octopus bend.

The most kinematically rigorous studies to date used bend with twist. Twist was reported in fewer studies because it required 3D data [7–9, 11]. Pairing bend and twist was a significant advancement in furthering kinematic decomposition of octopus movement, but it had one major drawback: it could only be used in arm postures where bending was directed toward the suckers so any time the arm was not in a single plane, it would be attributed to and quantified by a single angle twist. However, observational studies frequently saw arm bending movements that were away from the suckers, lateral, and anywhere in between [6]. These complex postures are part of what makes octopus movements unique and, therefore, require kinematic approaches that can comprehensively describe the entire posture of the arm in 3D space. Specifically, the complete set of relative rotation angles are needed between sections of the arm to define these complex motions.

For visualization of the arm in 3D, some researchers utilized markerless motion capture approaches and attempted to reconstruct the entire length and posture of the octopus arm; but this approach yielded significant challenges [1, 11]. Markerless motion capture suffered from long manual tracking periods that were often due to the inability to isolate the arm of interest, lack of bony landmarks, and the octopus’ camouflage ability [1, 10–14]. Additionally, studies that utilized these data were still limited by the kinematic approaches and posture constraints. Importantly, their complex movements, which require more rigorous kinematic approaches, are relevant to fields of study outside of the octopus.

The clinical field is an area that has the potential to utilize an array of movements in the form of octopus arm-inspired devices. Many current, soft, long, thin devices took the form of soft grippers [15–17]. The use of such devices in clinical settings ranged from assisting in surgery to a hand orthotic for help in grasping [16–18]. These flexible devices were often one segment or only capable of bending/twisting in one direction [16, 18]. Thus, methods for quantifying the posture of the device were as simple as the expected movements. However, octopus arm movements have the potential for different curvature magnitudes and directions on the nearly infinite number of segments along the arm; and with the advancement and anticipation of similarly capable soft devices, methods to quantify each segment with respect to another is a necessity.

It is also important to effectively communicate research findings to fields like health monitoring and rehabilitation, which are increasingly utilizing soft, orthotic devices. Currently, there are dynamic models for segmented, soft devices that can be used to predict the endpoint of a segment or series of segments [15, 16]. However, they require an intricate understanding of material properties and modeling, of which each unique device will have their own properties. Although this information is important for driving the endpoint of certain devices, it lacks translatability particularly with regard to health monitoring and rehabilitation outcomes. Octopus arm-like orthotics have the potential to track clinical outcome measures like joint rotations, spinal curvature and orientation, and muscle rigidity with the proper measurement and analysis approach. Such outcome measures could be used to track compensatory movements, scoliosis severity, tissue properties, and overall health [18, 19].

Thus, the primary goal of this work was to create a method to comprehensively quantify complex, 3D postures of octopus arms in a manner that is conducive and translatable to octopus arm-inspired devices for health monitoring and rehabilitation. Additionally, former approaches to quantify bend magnitude were compared and a discussion of the impact and use of this work in relation to future soft sensing devices will be presented.

## Methods

### Experimental methods and animal care

**Fig 1:**
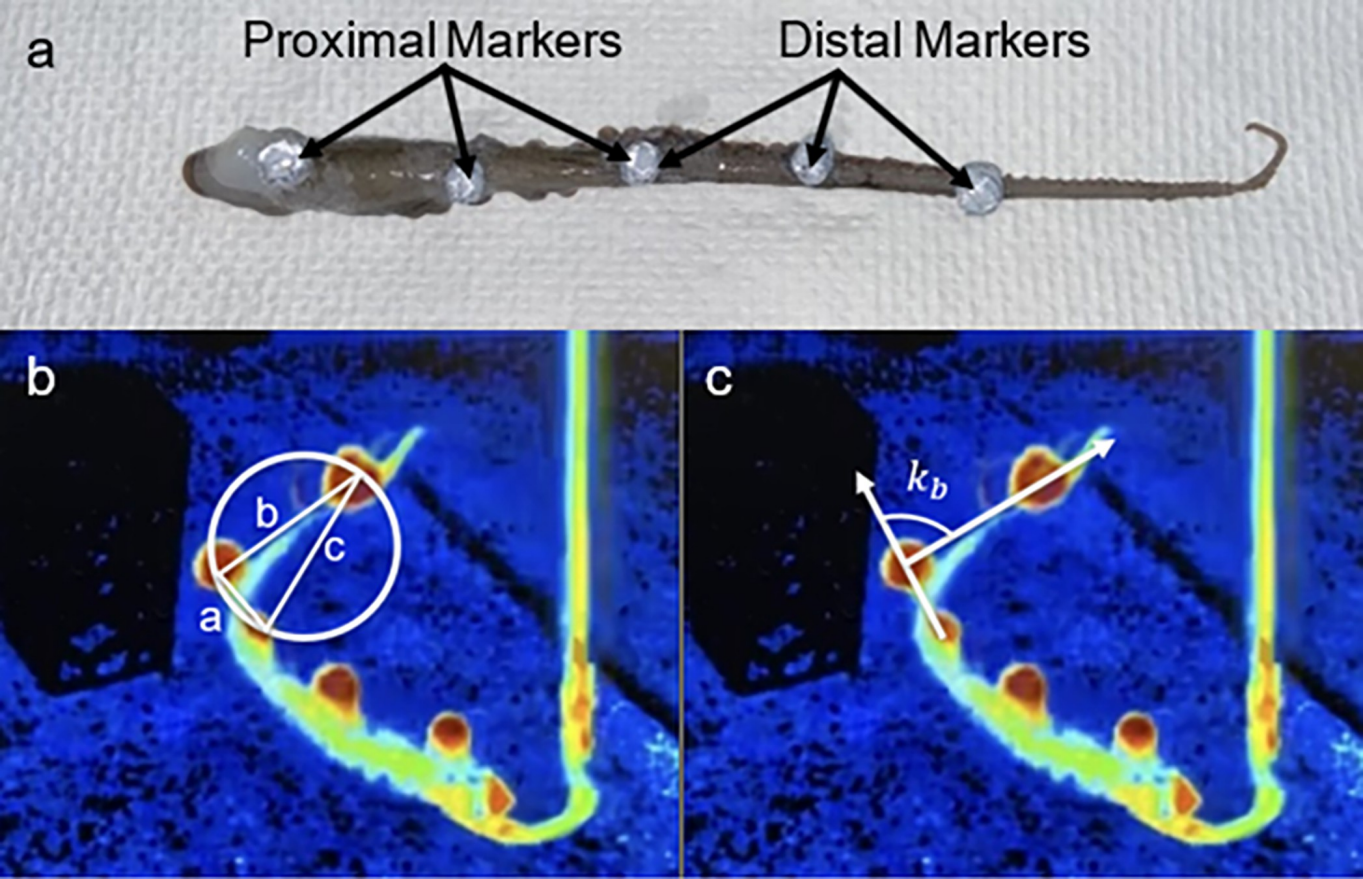
(a) Octopus arm marker attachment method. Five retroreflective markers were adhered to the arm. The proximal segment consisted of the first three markers. The distal segment consisted of the last three markers. The two methods for measuring curvature are also shown. (b) The distal segment of the arm depicts the radius of curvature (*k*_*o*_), calculated by the lengths of the sides (a,b, and c) between three points on the circle. (c) The distal segment of the arm depicts the vector curvature (*k*_*b*_), defined by the angle made by two consecutive vectors.

All experimental procedures were approved by the Michigan State University Institutional Animal Care and Use Committee. A four-camera underwater motion capture system (Qualisys, Gothenburg, Sweden) was used to capture 3D motion data of disembodied octopus arms that still had neural activity as well as live octopuses. Octopuses (*Octopus bimaculoides*) were from the California coast. For disembodied arm experiments, octopuses were anesthetized with 3% ethanol in a saltwater tank and arms were obtained using a blade to amputate at the proximal end. Octopuses with an amputation were given a dose of lidocaine to ease pain and discomfort after surgery and their health was monitored closely over the next 24–48 hours. Octopuses have the ability to regrow limbs so that progress was also tracked. If an octopus ever had two surgeries on different arms (for a different study), the animal was euthanized using a magnesium chloride solution. Disembodied arms were kept in saltwater that maintained neural activity of the axial nerve and motor activity in the arm. Five retroreflective markers were attached to the skin along the length of the arm using tissue adhesive (Vetbond by 3M, Minnesota, United States), and the arm was adhered to a short pole (Fig 1A). Then the pole was moved underwater to replicate the natural motions of the octopus arm, including twisting and bending in different directions. Experiments occurred underwater so the arm could experience similar resistance, and the saltwater environment was able to maintain some neural and motor activity.

A similar approach was taken for the live octopus experiments. An octopus was anesthetized with 3% ethanol. When the heart rate reached 20 beats per minute and the octopus did not respond to touch, the second arm on the right side (R2) was selected for marker attachment. The arm was held out of water, dried with Kimtech wipes (Kimtech Wipes by Kimberly-Clark, Neenah, Wisconsin, United States), and five markers ranging 3–5 mm in diameter were adhered to the dorsal side of the arm using the same tissue adhesive. Once all the markers were attached, the octopus was moved to a saltwater recovery tank where it was allowed to wake up. Once awake, the octopus was moved into the experimental tank where it was allowed to move freely. There were no exclusion criteria for the octopus in this study.

Positional data of the five markers located on the octopus arm (living or disembodied) were collected at a sampling rate of 150 Hz. Custom created software using MATLAB was developed to analyze the data. The positional data were used to compute 1) the curvature of different segments along the arm and 2) the planar orientation of one segment relative to another. These methods were applied to disembodied and live octopus arms and are described next.

### Analytical methods

An analysis was developed to describe the posture of the octopus arm in 3D. In these analyses, the arm was segmented into proximal and distal regions. The proximal segment consisted of the first three arm markers and the distal used the last three arm markers, of which the two segments shared the middle marker (Fig 1A).

Two approaches from literature were used and compared to analyze curvature. The goal of which was to compare the two techniques and conclude which would be more applicable to a human wearable device. The first approach used a triangle created by three consecutive position points. The sides of this triangle (*a*, *b*, and *c*) and half the perimeter were used to calculate the value for each segment’s curvature, called *k*_*o*_ in this manuscript (Eq 1). This value was equivalent to the inverse of the radius, *R*, of the circle passing through the three points (Fig 1B).


$$k_{o}=\frac{4\sqrt{s(s-a)(s-b)(s-c)}}{abc}=\frac{1}{R}$$ Eq 1

The second approach quantified curvature using an angle measurement made by two consecutive vectors ($\hat{v_{1}}$ and $\hat{v_{2}}$) (Fig 1C). The vector analysis resulted in (*k*_*b*_) values (Eq 2).


$$k_{b}={cos}^{-1}(\frac{\hat{v_{1}}∙\hat{v_{2}}}{\left|\hat{v_{1}}||\hat{v_{2}}\right|})$$ Eq 2

The two methods were applied to each segment of the arm. Thus, a curvature plot was produced for the proximal segment (using the first three markers), and the distal segment (using the last three markers). The advantages and disadvantages of both approaches were compared during key arm postures and will be discussed in the context of wearable devices.

Independently, neither of the above curvature approaches was sufficient to describe the entire rotational motions of the octopus arm. To do this, an additional analysis of the movement of one segment relative to another in all three planes was required. Non-planar movements occurred when the direction of the bend of the distal segment differed from that of the proximal segment. This happened either because arm segments were bending in different directions (for example the proximal segment was bending toward the suckers and the distal segment was bending away from the suckers) or that a twist occurred along the arm and changed the plane of curvature (for example, both segments could be curving toward that segment’s suckers, but a twist would change the planar orientation between the two segments). Kinematically defining the direction of curvature was a necessary feature to completely quantify complex arm motions of the octopus.

**Fig 2:**
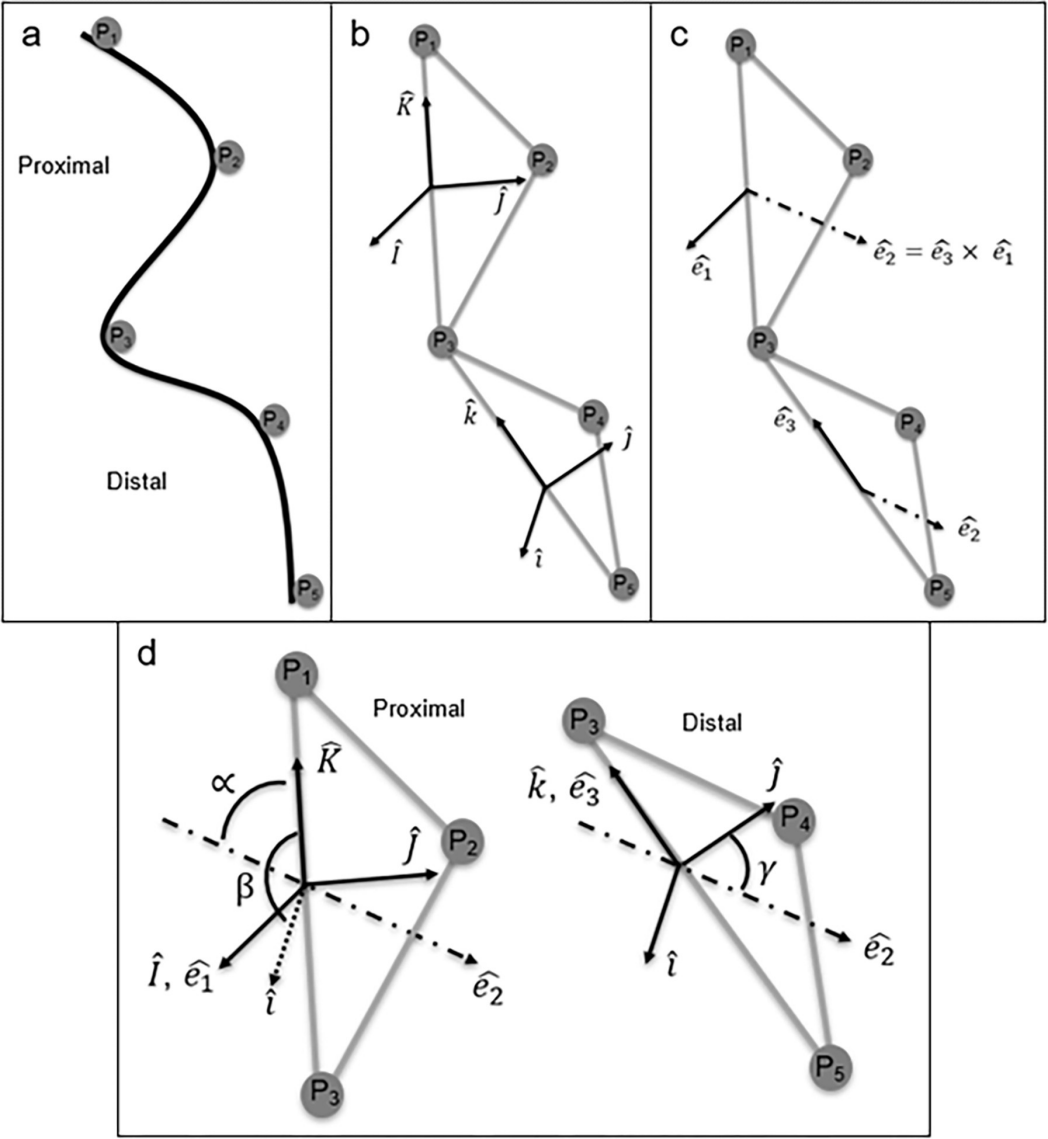
(a) Markers used to create two distinct local coordinate systems and describe the overall posture of the octopus arm. (b) The upper-case coordinate system ($\hat{I},\hat{J},\hat{K}$) was created on the proximal segment and the lower-case coordinate system ($\hat{i},\hat{j},\hat{k}$) was created on the distal segment. (c) Additionally, fixed body and floating axes were created using the two segments. Octopus arm posture was shown such that measurements would result in differences in planar orientation. (d) Differences were reported using three rotations: ∝ representing flexion/extension, *β* representing abduction/adduction, and *γ* representing internal/external rotation.

In order to measure the differences in planar orientation between the two segments, the arm was segmented as it was for curvature (Fig 2A). Two local, orthogonal coordinate systems (LCS) were created: one on the proximal segment using the first three markers and one on the distal segment using the last three markers. The creation of these LCSs followed the method by Grood and Suntay [20]. The proximal LCS was denoted by capital letter unit vectors ($\hat{I},\hat{J},\hat{K}$), whereas the distal LCS was denoted by lower case unit vectors ($\hat{i},\hat{j},\hat{k}$) (Fig 2B). Vectors for the proximal and distal arm segments were created similarly. $\hat{K}$ and $\hat{k}$ vectors were created using the first and third markers of their respective segment (*P*_1_ and *P*_3_ denoted in the proximal segment, and *P*_3_ and *P*_5_, denoted in the distal segment). $\hat{I}$ and $\hat{i}$ were the vectors normal to the plane and were obtained by taking the cross product of the vector created by the respective second and third markers (*P*_2_ and *P*_3_, and *P*_4_ and *P*_5_) and the vector $\hat{K}$ (or $\hat{k}$). The last LCS vectors, $\hat{J}$ and $\hat{j}$, were created by taking the cross product between $\hat{K}$ (or $\hat{k}$) and $\hat{I}$ (or $\hat{i}$). These vectors were normalized to a length of 1 and created the LCS for the two arm segments. The following calculations were used for both segments, but only the equations for the proximal LCS axes computations are shown (Eqs 3–6).


$$\hat{K}=\frac{P_{1}-P_{3}}{\left|P_{1}-P_{3}\right|}$$ Eq 3

$$\vec{v_{1}}=P_{2}-P_{3}$$ Eq 4

$$\hat{I}=\frac{\vec{v_{1}}\times \hat{K}}{\left|\vec{v_{1}}\times \hat{K}\right|}$$ Eq 5

$$\hat{J}=\frac{\hat{K}\times \hat{I}}{\left|\hat{K}\times \hat{I}\right|}$$ Eq 6

Before the relative plane angles between the two planes could be determined, three more axes were defined: ${\hat{e}}_{1},{\hat{e}}_{2}$, and ${\hat{e}}_{3}.\;{\hat{e}}_{1}$ and ${\hat{e}}_{3}$ were fixed body vectors, which were equivalent to the previously defined $\hat{I}$ and $\hat{k}$, respectively. ${\hat{e}}_{2}$ was defined as a floating axis, which was determined by the cross product between ${\hat{e}}_{3}$ and ${\hat{e}}_{1}$ (Eqs 7–9) (Fig 2C).


$${\hat{e}}_{1}=\hat{I}$$ Eq 7

$${\hat{e}}_{3}=\hat{k}$$ Eq 8

$${\hat{e}}_{2}=\frac{{\hat{e}}_{3}\times {\hat{e}}_{1}}{\left|\hat{{\hat{e}}_{3}}\times {\hat{e}}_{1}\right|}$$ Eq 9

Planar orientation was then measured using the LCSs, the fixed body axes, and the floating axis. In human biomechanics and clinical settings, these rotations have been called flexion/extension, abduction/adduction, and internal/external rotation (Eqs 10–12) [20].


$$Flexion(‐)/extension(+):\;\sin\left(\propto \right)=-{\hat{e}}_{2}∙\hat{K}$$ Eq 10

$$Abduction(+)/adduction(‐):\;\cos\left(\beta +\frac{\pi }{2}\right)={\hat{e}}_{1}∙{\hat{e}}_{3}$$ Eq 11

$$External(+)/internal(‐)rotation:\;\tan\left(\gamma \right)=\frac{n{\hat{e}}_{2}\times \hat{j}}{{\hat{e}}_{2}∙\hat{j}}$$ Eq 12

The negative sign associated with α and the $+\frac{\pi }{2}$ value associated with β were a result of using a right-handed mathematical system. Since this analysis was primarily applied to the human body within the literature, the right side of the body determined how positive values of flexion, abduction, and external rotation were defined. In this analysis, the right side of the body system was used, as outlined by Grood and Suntay [20]. Additionally, the definition for α was limited to ±90° and the definition for β limited to 0 to 180°. Because it was not possible for human segments to reach more than 180° of movement for α and β, sine and cosine definitions were sufficient. However, because γ could reach values ranging from 0 to 360° for an octopus, sine and cosine alone were not sufficient. These equations were modified to utilize a tangent function. Tangent was used with a vector *n* (unit vector that determined the quadrant) to measure the full 360° of potential external/internal rotation.

There are, however, distinct differences between the methods applied in this study and those offered by Grood and Suntay in 1983. First, and most obvious, is that the Grood and Suntay method (along with other rigid body rotation approaches like Euler and Cardan) were applied to rigid bodies. In this study, the methods were applied to soft bodies. The effects of this were twofold. First, LCS vector notation and direction were arbitrary and could not be directly translated from Grood and Suntay to the octopus arm. To address this, $\hat{K}$ vectors were made using the direction of the ‘straight’ posture (connecting the first and third markers on the segment), $\hat{I}$ vectors were created by identifying the outward direction of the bend from the $\hat{K}$, and finally $\hat{J}$ by taking the logical cross product. These definitions resulted in similar properties for rigid body rotations (flexion, abduction, internal rotations observationally in the correct directions). The second effect was that the three orientation angles do not represent motion at any specific ‘joint’ on the octopus arm, rather just how two segments are oriented with respect to each other. In soft devices with clearly defined segments, this would not be an issue. However, for octopus arms with no pre-defined segments, this must be taken into consideration.

### Methods summary

Definition and measurement of segment curvature and plane orientation were combined to create a novel method for a quantification of octopus arm posture in 3D space for use with soft, wearable devices. It also offered a way that could be implemented and communicated within and between biological, biomechanical, and clinical fields. Three octopus arm postures were analyzed, including a nearly straight, an in-plane bending (also known as simple bending), and a complex bending scenario, since previously, only kinematically simpler movements were able to be analyzed. Thus, for each posture, for both the nine disembodied arms and the living octopus, proximal and distal segment curvatures will be reported for the two methods (*k*_*o*_ and *k*_*b*_) as well as the planar orientation differences between the two segments (∝ for flexion/extension, *β* for abduction/adduction, and *γ* for internal/external rotation). The straight posture was visually identified in the motion capture system as the point of minimum overall curvature, and where the proximal and distal segments were most in line with each other. The simple bending posture was identified when there was maximum overall curvature and the octopus arm laid primarily on one plane. Finally, complex curvature postures were identified when the proximal and distal segments had clearly different orientations.

## Results

### Disembodied octopus arm results

**Fig 3:**
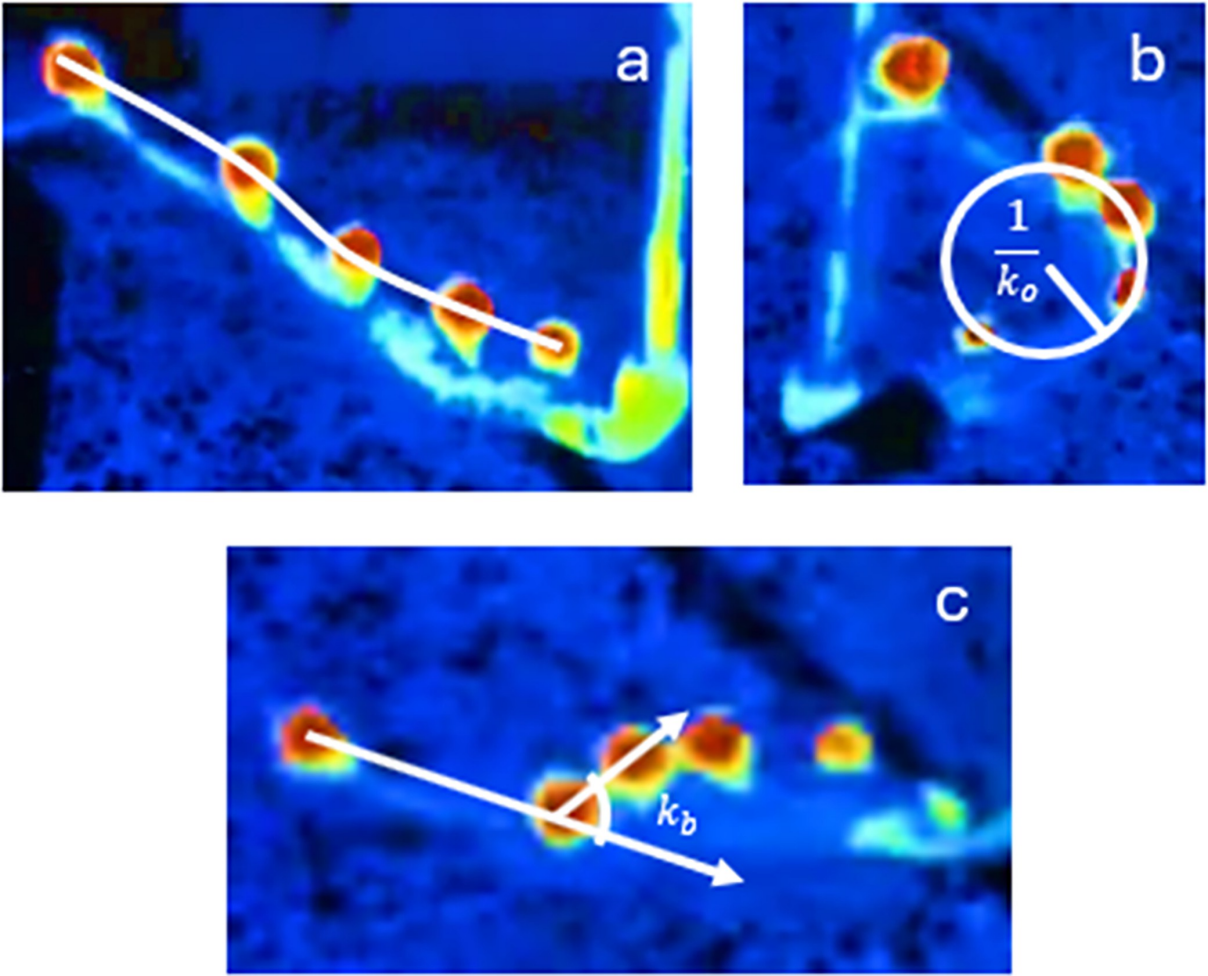
(a) Octopus arm in nearly straight posture: white line indicates approximate ‘backbone’ of markers. (b) Octopus arm in simple bending posture, where the arm seemingly lays on one plane: white circle depicts again how *k*_*o*_ is calculated. (c) Octopus arm in complex bending posture, where the arm is not on one plane: white vectors depict again how *k*_*b*_ is calculated.

To compare the different curvature approaches, three postures were analyzed using *k*_*o*_ (the radius of curvature approach) and *k*_*b*_ (the vector curvature approach) (Fig 3A and 3B, respectively). An example of one octopus arm in the three postures is shown in Table 1. This is done to highlight the differences in curvature approaches.

**Table 1:** Outcome measures for proximal and distal arm segments in the three postures as demonstrated through one of the test trials (n = 1): Nearly straight, simple bending, and complex bending which are shown in Fig 3. The approach using *k*_*o*_ is strongly influenced by segment length in comparison to *k*_*b*_ approach.

Posture	Proximal *k*_*o*_ (mm^-1^)	Proximal *k*_*b*_(°)	Distal *k*_*o*_ (mm^-1^)	Distal *k*_*b*_(°)
Nearly Straight	0.007	8.4	0.004	8.7
Simple Bending	0.043	64	0.016	36
Complex Bending	0.024	33	0.019	46

In this example of one disembodied arm, the three postures resulted in different curvature values for each segment in this example octopus arm (Table 1). The nearly straight posture, as expected, had the least amount of curvature in comparison to the simple and complex bending postures, regardless of the approach used. Yet, there was one main difference between *k*_*b*_ and *k*_*o*_, and that was their sensitivity to segment length, which was highlighted by the curvature results of the straight posture. Although the *k*_*o*_ metric yielded drastic differences in curvatures of 0.007 mm^-1^ and 0.004 mm^-1^ for the two segments, the *k*_*b*_ values were much closer (8.4° and 8.7°) for the same two segments. Because of the varying segment lengths, it was possible to obtain a different *k*_*o*_, yet have a similar *k*_*b*_ value for the two segments. Since this work will be applied to human wearables and biomechanics, the vector approach (*k*_*b*_) was determined to be the most appropriate because of the approach’s decreased sensitivity to segment length. Because of this, the curvature will be reported using *k*_*b*_ only.

**Fig 4:**
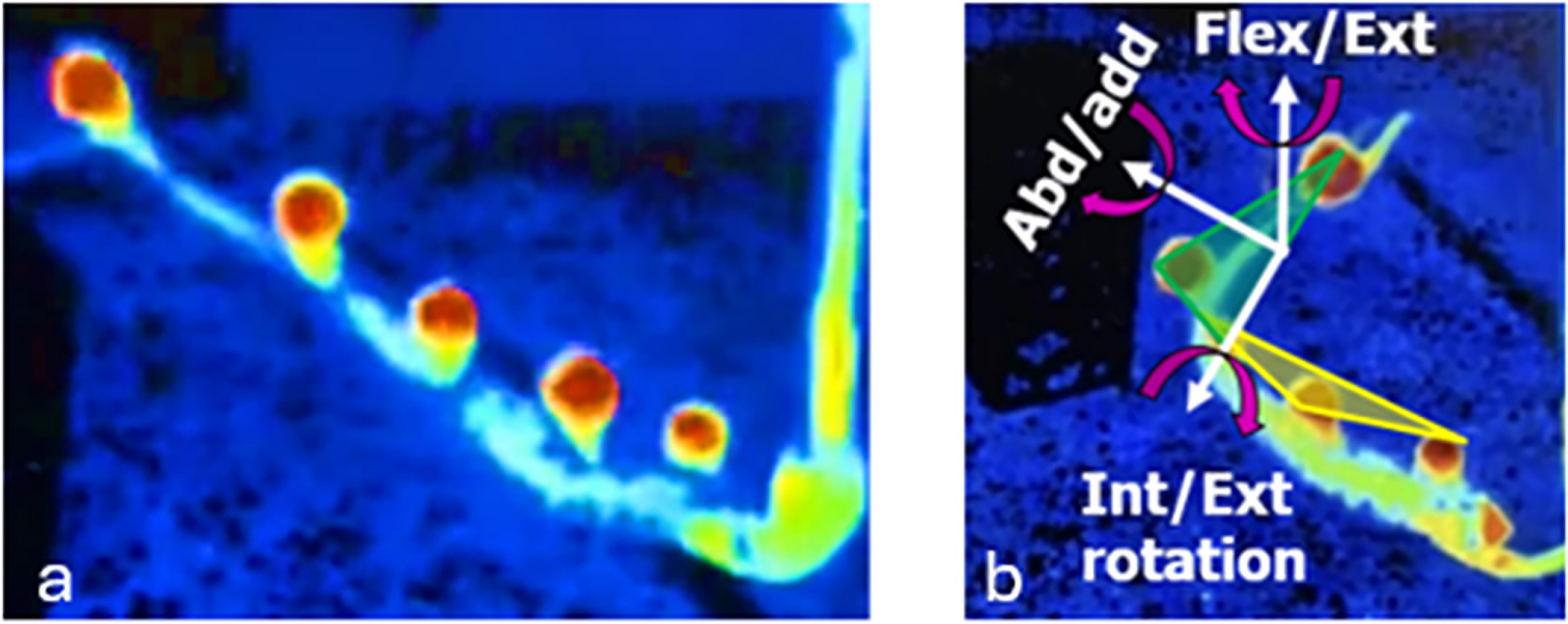
(a) Octopus arm in straight posture for reference. (b) Octopus in simple bending posture: local coordinate systems are shown in yellow and green for the proximal and distal segments, respectively. Vectors show 3D rotations. Since simple bending appears to have both segments on similar planes, it is expected that the largest contribution to orientation difference will be in the flexion/extension direction.

Next, the full kinematic analysis (magnitude of curvature and planar orientation for each segment) was applied to the nine disembodied arms during the nearly straight and simple bending postures (Table 2). These two postures (Fig 4) were of focus for the disembodied arms because they were easily repeatable underwater, in contrast to complex bending postures. These were also the only postures that could be approximately quantified using previous approaches.

**Table 2:** Average (standard deviation) proximal curvature, distal curvature, and planar orientation values for all disembodied arms (n = 9) while being moved underwater. The simple bending posture (Fig 4B) consistently had larger proximal curvatures, distal curvatures, and planar orientation differences between proximal and distal segments. Although perfectly planar postures would be expected for observationally straight and simple bending, non-zero values for abduction and internal rotation were measured, indicating that the arm was not in as simple a posture as expected.

Posture	Average proximal *k*_*b*_ (°)	Average distal *k*_*b*_ (°)	Average flexion (°)	Average abduction (°)	Average internal rotation (°)
**Nearly Straight**	25.7 (20.1)	7.2 (5.2)	6.6 (11.8)	1.4 (10.2)	0.7 (6.5)
**Simple bending**	55.7 (21.9)	47.5 (15.2)	79.0 (11.9)	16.8 (32.6)	-11.8 (22.7)

The largest curvature values for the proximal and distal segments occurred when the posture was in the simple bending condition and, on average, were 30.0° and 40.3° greater than those segments during the straight posture (Table 2). The straight posture resulted in flexion, abduction, and internal rotations that were close to 0° (on average 6.6°, 1.4°, and 0.7° respectively). This meant that there was little difference in alignment between the proximal and distal segments, but there was still some even given the observational collinearity of the markers. The simple bending posture resulted in abduction and external (negative) rotation values greater than the straight posture (on average 16.8° and 11.8°). However, there was a significant spike in the flexion value (on average 79°), which was attributed to that characteristic bending of the octopus arm. Complex bending scenarios were excluded for the disembodied arms because movements were not able to be repeated for analysis of multiple trials, so they will be discussed next, only for the live octopus.

### Live octopus results

**Fig 5:**
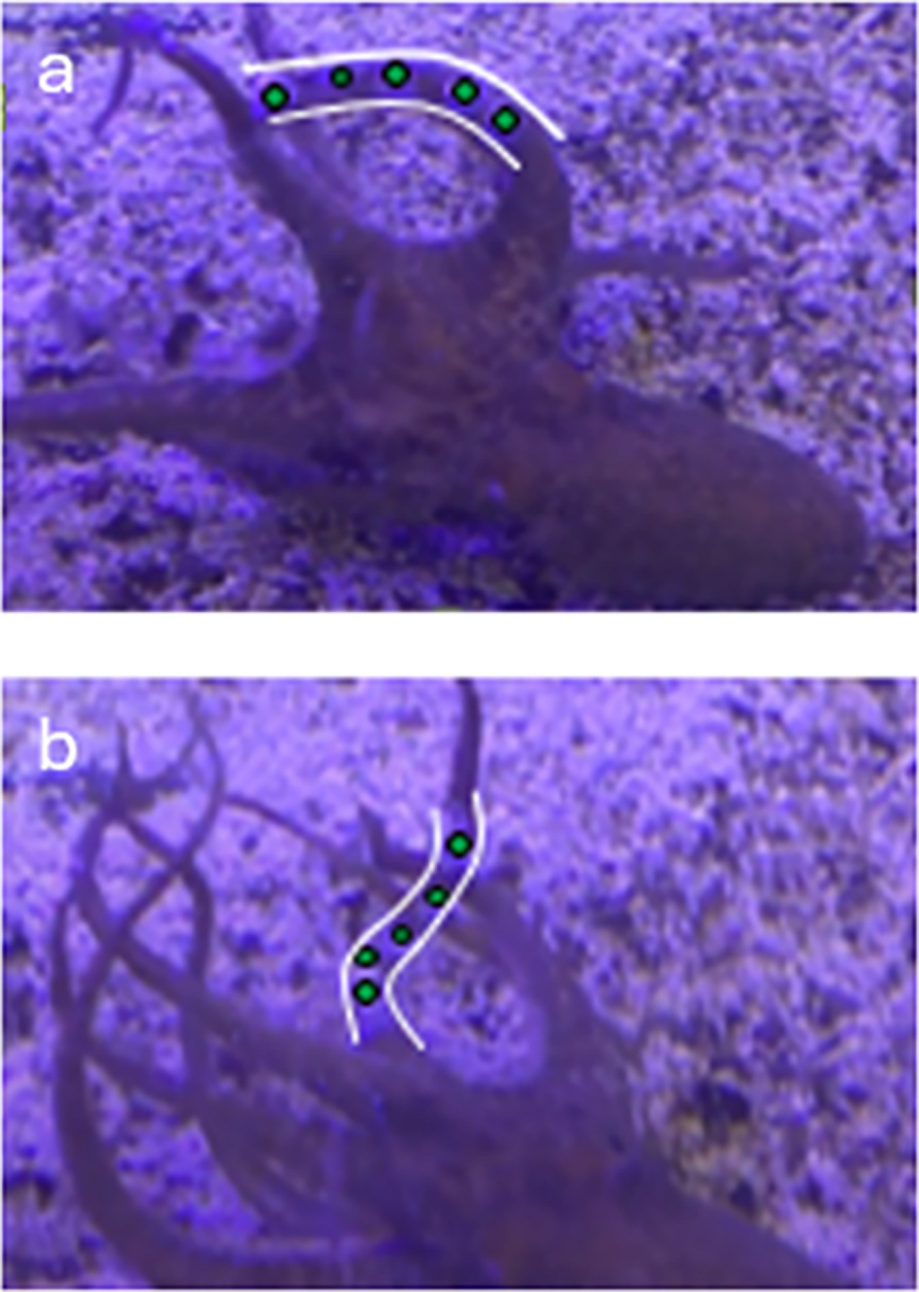
Live octopus exhibiting (a) simple bending posture, where markers appear to lay on the same plane and (b) a complex bending posture, where markers are not in the same plane, due to bending in different directions along with complex rotations. In both images, the white curves highlight the arm of focus and the green circles indicate the marker locations.

The live octopus had movements that ranged from simple bending (Fig 5A) to complex bending (Fig 5B). The kinematic results of these postures follow (Table 3).

**Table 3:** Curvature and planar orientation results for a (n = 1) live, swimming octopus, also seen in Fig 5. Similar abduction and internal rotation data were obtained during the live octopus and the disembodied arm trials. The complex bending scenario had a large, negative internal rotation value, which suggests that the orientation of the distal segment was nearly flipped (145°) in comparison to the proximal segment.

Posture	Proximal *k*_*b*_ (°)	Distal *k*_*b*_ (°)	Flexion (°)	Abduction (°)	Internal rotation (°)
**Simple Bending**	27	20	28	19	13
**Complex Bending**	33	12	-25	-11	-145

The live octopus results demonstrated the need for this new approach; even during what appears to be simple bending postures from visual observations, there were relative rotations occurring between segments. Additionally, the complex bending posture seen above is something that could not be quantified using previous approaches. By having a tool that can quantify 1) the magnitude of curvature on each segment and 2) the orientation of one segment relative to the previous (i.e., the direction of the curvature), any number of arm postures can be tracked.

## Discussion

### Kinematic discussion

Prior research documenting octopus arm movements faced a number of challenges. First, there were inconsistencies in how bending was reported. The results of both approaches reflected deviations from the straight posture with increased curvature values, but the radius of curvature approach (*k*_*o*_) was more heavily influenced by segment length. Additionally, the vector approach (*k*_*b*_) is more like outcome measures already used in clinical applications, such as joint rotations and spine curvature [21–23]. Therefore, the vector approach was deemed more appropriate in translation to clinical devices.

The second issue with previous studies was with the constraints that the kinematic methods relied upon, such as planar movements or bending that could only occur toward the suckers [1, 11]. Even postures that were expected to have very little out of plane movement still required the use of the three rotations to orient one segment relative to the other. Curvature and single twist kinematic definitions used by prior researchers would not cover complex movements, like segments bending in different directions [7, 11].

Also, the comprehensive kinematic approach in this study was developed for application to segmented soft devices and supports clinical translation. Devices inspired by muscular hydrostats, like snakes and octopods, are relatively new to the field. Initial devices are being explored by researchers to replace rigid devices because soft devices offer greater degrees of freedom and are capable of unique movements and postures, while offering simpler and cost effective designs [15, 24–26]. Though recent devices ranged in function from picking up objects to assisting in surgery, functional designs have been similar in that they were often only one segment or were multi-segmented but could only bend in one direction [15, 24, 27]. It is anticipated that as research advances, more intricate systems (involving multiple, independent segments), capable of complex postures and higher degrees of freedom will be developed, for a wide array of applications [28].

Smart orthotics, remote patient monitoring, and in-person rehabilitation often rely on outcome measures to inform diagnoses and treatment paths [29, 30]. For example, monitoring changes in the spine as a result of scoliosis, have relied on 2D X-Rays [21, 31]. The ability to dynamically monitor with a flexible, wearable device would significantly advance evaluation of the effectiveness of therapy or other treatments. A segmented, flexible, orthotic device could provide real-time information on the magnitude of curvature of each segment of the spine and the location or direction of that curvature. Another application of such a device is in retraining of movements during rehabilitation. Already a powered, flexible hand orthosis exists that can assist in 2D motion of flexion and extension of the finger joints [18]. However, more complex devices are likely to be developed; one possibility is an octopus arm-inspired orthoses, using curvature and orientation, which would be flexible enough to contour to any part of the body and assist regardless of the complexity of the joint. Thus, these devices could help the body through compensatory movements that are outside of normal ranges or types of motions such as those found in Parkinsons’ Disease and post- stroke [32–34]. Additionally, such a device does not need to only be applied to the rigid, linked systems of the human body; future developments may permit the tracking of soft tissue deformations. For example, an expandable, soft orthotic on the thigh and buttocks in the seated position could potentially provide information on tissue degeneration or bony protrusions that when they interface with the seat lead to increased pressure injury risk. Overall, the use of the methods and results of this study have the potential to support an array of new device types that have the ability to expand upon existing outcome measures that are crucial for health monitoring in the clinical field.

### Future work discussion

The findings of this paper can be used to complement other sensing systems like accelerometers or strain gauges for flexible devices [35–37]. Complex deformation sensing is a rich field in soft devices and there exist useful techniques that can pair with the kinematic octopus results for effective health monitoring. Piezoelectric sensors and strain gauges have been used in determining soft tissue strain in humans and bio-inspired devices [38–40]. Such technology could be used to determine magnitude and direction of curvature at local points (segments) of soft devices and motion capture approaches could be used to validate those associated new measurements in a laboratory setting. Then, when on the person as a soft, wearable, outside of the lab and a motion capture system, these alternative sensors could provide the bend and orientation measures. Thus, this approach can complement others sensing systems. The outcome measures of such future systems have the potential to provide information such as tissue properties, compensatory movements, and spinal postures. Other forms of sensing through accelerometers could also provide information on the overall posture of the bio-inspired devices [41]. Additionally, the approaches in this study can be used for complex octopus movements in understanding areas of muscle activation, trajectory generation and model development. Fine wire electrodes have been used on disembodied octopus arms to map muscle activation and it is likely a similar capability will soon be available for living octopuses [42]. Thus, the findings of this paper can provide significant advancements with current clinical challenges when partnering with soft devices, but they also have the potential to foster, new devices and complement sensing systems in rehabilitation and other health monitoring.

### Limitations

The use of optical motion capture for data collection had challenges such as marker adherence to the arm and octopuses inking the tank which resulted in blocked camera views; however, these were overcome for this work. Some researchers explored the use of markerless motion capture, but this approach yielded significant challenges. Markerless motion capture suffers from long manual tracking periods that are often due to the inability to isolate the arm of interest, lack of bony landmarks, and the octopus’ camouflage ability [1, 10–14]. As an example, one simple 2D, human study, found that joint landmarks needed to be tracked between 300 and 400 times to properly train a model and noted that training to accuracy similar to that of optical motion capture took 9–12 hours [43]. It is expected, that since octopuses have complex, 3D movements that the time associated with tracking/training would require even more effort than this basic human study [1, 11–14]. Because of this, optical marker motion capture has fewer challenges for tracking in comparison to markerless approaches.

Other limitations were in the mathematics of the analysis; in the unlikely scenario where three markers on the proximal segment were exactly colinear the mathematical definition would not work. This was because there would be no way to create a local coordinate system and the necessary axes. However, this condition is unlikely in a swimming or moving octopus arm and is also a limitation of the previous approach utilizing the single angle twist.

### Significance

In conclusion, a comprehensive method for quantifying octopus kinematics was developed and implemented on both disembodied arms and living octopus to determine the overall three-dimensional posture of the arms. This included successfully segmenting the arm, mathematically computing curvature on a given segment, and determining the relative orientation of segments. This together permitted the quantification of complex octopus arm motions. In addition, this new method supports the development of new soft wearable devices, trajectory mapping of complex motions, and complex deformation mapping; and it has the potential to be used in concert with other multi-modal measurement tools. The impact of this work is far-reaching, particularly in the area of device development for rehabilitation and health monitoring.

## Supporting information

S1 File Disembodied octopus arm curvature and planar orientation results. (DOCX)

S2 File ARRIVE 2.0 checklist. (PDF)

S3 File MATLAB orientation function. (M)
