# Supplementary material for: 3D octopus kinematics of complex postures: Translation to long, thin, soft devices and their potential for clinical use
Source: PLoS One. 2024 May 29;19(5):e0303608. doi: 10.1371/journal.pone.0303608 (PMC11135735; doi:10.1371/journal.pone.0303608)
Supplement: S1 File — (DOCX) [file pone.0303608.s001.docx]

Supporting Information Document

This document includes additional data and information pertaining to the reported curvature and planar orientation results. As described, a varying level of posture complexity was analyzed using the two approaches – of which the averages were reported for the disembodied arms and certain time points were reported for the live octopus. Supplemental Figure A shows the curvature and planar orientation plots for one disembodied octopus arm being moved straight, turning around, and straight again.


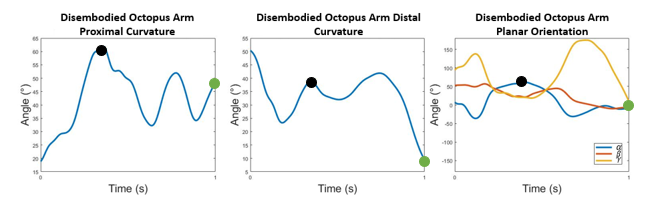


**Supplemental Figure A. Stereotypical profiles for disembodied octopus arms while going straight, turning around, and continuing straight. The black circles (all located at the same point in time) indicate when the octopus arm reached maximum curvature and was identified as simple bending – characteristic of this point was maximum local curvature values (left, middle) and minimized β and γ values (right). The green circles (all located at the same point in time) indicate when the octopus arm was identified as straight – characteristic of this point was lower curvature values (left, middle) and minimized values for all planar orientation angles (right).**

Averaged for all nine arms, the straight and simple bending postures resulted in the following results (Supplemental Table A and B). These values were used to calculate the averages presented in Tables 1 and 2 in the manuscript.

**Supplemental Table A. Straight posture curvature and planar orientation values obtained for all nine disembodied octopus arms.**

| Disembodied octopus arm # | Proximal $k_{b}$ (°) | Distal $k_{b}$ (°) | Flex./Ext.  $\propto$ (°) | Abd./Add.  $\beta$ (°) | Int./Ext. Rotation $\gamma$ (°) |
| --- | --- | --- | --- | --- | --- |
| 1 | 32.7 | 7.6 | -6.0 | -13.5 | -2.0 |
| 2 | 21.9 | 7.5 | -6.4 | 12.8 | -6.4 |
| 3 | 61.1 | 6.3 | 26.3 | 1.2 | 1.2 |
| 4 | 19.2 | 19.7 | 0.7 | 10.5 | 10.5 |
| 5 | 19.0 | 8.1 | -2.2 | 1.0 | -0.6 |
| 6 | 54.7 | 1.7 | 18.6 | -4.6 | 0.7 |
| 7 | 4.1 | 6.3 | 13.9 | 13.9 | 4.1 |
| 8 | 8.7 | 2.0 | 14.0 | 4.0 | 8.4 |
| 9 | 10.0 | 5.7 | 0.8 | -12.9 | -10.0 |

**Supplemental Table B. Simple bending posture curvature and planar orientation values obtained for all nine disembodied octopus arms.**

| Disembodied octopus arm # | Proximal $k_{b}$ (°) | Distal $k_{b}$ (°) | Flex./Ext.  $\propto$ (°) | Abd./Add.  $\beta$ (°) | Int./Ext. Rotation $\gamma$ (°) |
| --- | --- | --- | --- | --- | --- |
| 1 | 46.1 | 43.0 | 86.8 | 36.5 | -29.2 |
| 2 | 61.3 | 38.7 | 57.7 | 23.9 | 22.3 |
| 3 | 87.4 | 76.8 | 86.7 | 46.4 | -24.0 |
| 4 | 42.5 | 54.0 | 88.7 | -36.0 | -3.8 |
| 5 | 31.1 | 34.8 | 74.0 | -1.5 | -42.5 |
| 6 | 88.8 | 50.1 | 88.3 | 64.7 | -29.2 |
| 7 | 51.9 | 63.7 | 89.3 | 29.7 | 12.2 |
| 8 | 64.0 | 36.0 | 64.2 | 10.5 | 11.3 |
| 9 | 28.1 | 30.4 | 75.3 | -22.6 | -22.6 |
